# Lactose Maldigestion, Malabsorption, and Intolerance: A Comprehensive Review with a Focus on Current Management and Future Perspectives

**DOI:** 10.3390/nu10111599

**Published:** 2018-11-01

**Authors:** Filippo Fassio, Maria Sole Facioni, Fabio Guagnini

**Affiliations:** 1 Allergy and Clinical Immunology Unit, Azienda Usl Toscana Centro, Ospedale San Giovanni di Dio, 50143 Firenze, Italy; 2 AILI—Associazione Italiana Latto-Intolleranti Onlus, 55100 Lucca, Italy; presidente@associazioneaili.it; 3 Allergy Therapeutics Italia, via IV Novembre 76, 20190 Milano, Italy; fguagnini@libero.it

**Keywords:** lactose intolerance, lactose maldigestion, lactose malabsorption, probiotics, food intolerance

## Abstract

Milk is a fundamental component of the diet of every mammal; nevertheless, not every individual can tolerate this kind of food, especially in adulthood. However, lactose intolerance has only been recognized in the last 50 years, and currently, lactose intolerance is defined as a clinical syndrome characterized by pain, abdominal distention, flatulence, and diarrhoea that occur after lactose consumption. Lactose is currently a common disaccharide in human nutrition, both in breastfed infants and in adults, but its digestion requires a specialized enzyme called lactase. The genetically programmed reduction in lactase activity during adulthood affects most of the world’s adult population and can cause troublesome digestive symptoms, which may also vary depending on the amount of residual lactase activity; the small bowel transit time; and, especially, the amount of ingested lactose. Several diagnostic tests are currently available for lactose intolerance, but the diagnosis remains challenging. The treatment for lactose intolerance mainly consists of reducing or eliminating the dietetic amount of lactose until the symptoms disappear, but this is hard to achieve, as lactose is present in dairy products and is even commonly used as a food additive. In addition to dietetic restriction of lactose-containing foods, lactase can be administered as an enzymatic food supplement, but its efficacy is still controversial. Recently, probiotics have been proposed for the management of lactose intolerance; certain probiotic strains have shown specific β-galactosidase activity, thus aiding in the digestion of lactose. The aim of this paper was to review the current knowledge about lactose intolerance and to discuss the potential for the use of specific probiotic strains such as dietary supplements in lactose-intolerant patients.

## 1. Introduction

Milk is a fundamental component of the human diet, and its nutritive value has been proved by a thousand years of constant use in human nutrition [1]. It is a specific type of food unique to mammals, which is the only nourishment for mammalian infants in the first months of life.

Milk, in addition to proteins, fats, vitamins, and minerals, contains carbohydrates composed of lactose and other important oligosaccharides that support the development of probiotic bacteria, in particular, *bifidobacteria*, in the infant’s intestine, to protect the child’s gastrointestinal tract from infections. However, it is also a complete food for adults, since it lacks only certain vitamins and iron [1].

Nevertheless, not everyone can tolerate this kind of food, especially in adulthood. Lactose intolerance is a clinical condition that has been known since the age of Hippocrates (460–370 BCE) and Galen (129–200 CE), who noticed that some individuals presented symptoms of gastrointestinal diseases after drinking milk [2,3]. Nonetheless, it has just been in the last 50 years that lactose intolerance has been recognized and scientifically analysed [2].

Currently, lactose intolerance is defined as a clinical syndrome characterized by pain and abdominal distention, flatulence, and diarrhoea that occurs after lactose consumption; this has to be distinguished from lactose maldigestion (inefficient digestion of lactose due to lactase deficiency) and malabsorption, which can also be subclinical conditions [3].

## 2. Molecular Mechanism of Lactose Maldigestion and Malabsorption

Milk contains a particular type of sugar: lactose. Lactose is a disaccharide only found in mammalian milk and in its derivatives, since it is synthesized in the mammary gland by the lactose synthetase system, which binds a D-galactose molecule to a D-glucose molecule with a β-1,4 glycosidic bond [1].

Lactose is such a common disaccharide in human nutrition that we often forget how “exotic” a compound it is; apart from dairy products, we can find it only in extremely rare plant species [4].

From the biochemical point of view, lactose metabolism presents some interesting peculiarities; human galactosyltransferase (subunit A of lactose synthetases) does not have enough affinity for glucose to allow lactose synthesis to occur. It needs subunit B for the enzyme to accept glucose and in women; hormonal adjustments (increase in prolactin and decrease in progesterone) have to occur to allow lactose synthesis [4].

**Figure 1:**
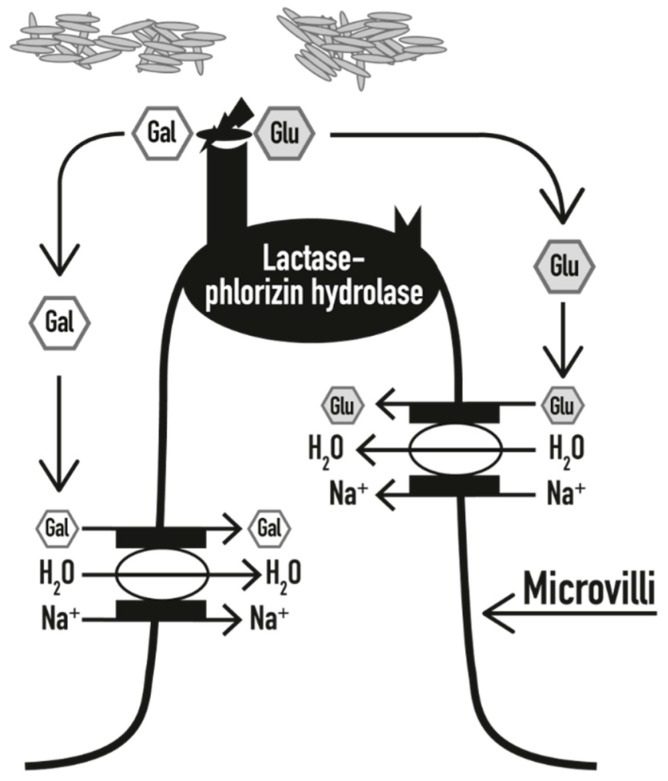
In the presence of an adequate amount of lactase, lactose is hydrolysed into galactose (Gal) and glucose (Glu), which are rapidly absorbed into the bloodstream, together with H_2_O molecules (modified from [5]).

Lactose digestion needs a specialized enzyme, commonly called lactase. Lactase is a β-galactosidase that can be found on the upper surface of enterocytes on the microvilli of the small intestine, and it is maximally expressed in the medium jejunum (where the bacterial concentration is low and, therefore, little fermentation occurs). It hydrolyses a lactose molecule in two monosaccharides, glucose and galactose, which, upon digestion, are rapidly absorbed by enterocytes and then used; the glucose is used as a source of energy, while the galactose is used as a part of glycoproteins and glycolipids (Figure 1) [1]. In the case of lactase deficiency, the disaccharide is not properly digested (lactose maldigestion) and therefore cannot be absorbed in an undigested form (lactose malabsorption) and is fermented by the gut microbiota.

The expression of lactase is programmed to change over time; already in the eighth week of gestation, lactase is present all over the mucous membrane of the small intestine; then, its activity increases until the 34th week and reaches its peak at birth [6]. After the first few months of life, the lactase activity begins to decrease, sometimes until it disappears. In humans, a considerable part of the population maintains lactase activity for all of adulthood, whereas in other mammals, maintaining lactase activity is unusual [6].

Indeed, the domestication of cattle promoted milk as a food item for adult nutrition. This was only made possible by two additional key factors, namely, the concomitant domestication of lactic acid bacteria, which ferment the non-digestible lactose to easily absorbed lactic acid, and the mutation to lactase persistence in adults from dairy societies [4]. This latter phenomenon occurred especially in Northern European populations and their heirs, who have a very low rate of lactose intolerance compared to Southern European or Asian populations [7].

It should be emphasized that, from the functional point of view, for adequate lactose digestion, the presence of 50% of the normal lactase activity is enough [1].

## 3. Genetic and Biochemical Background

From the zoological point of view, mammals are distinguished by two typical features, the presence of hairs, which provide thermal insulation, and the production of milk [8].

The mammalian classification itself actually refers to the mammary gland. Breastfeeding has many advantages compared to other methods of nourishment, since milk is a kind of food that has developed to provide the best nutritional support for the infant [4].

The small intestinal enzyme lactase (also called lactase-phlorizin hydrolase) is made of 1927 amino acids, is encoded by the gene lactase (*LCT*) on the short strand of chromosome 2 (2q.21-22) [9], and has two enzymatic activities: a lactase hydrolase activity and a phlorizin hydrolase activity. Lactase is responsible for the hydrolysis of lactose into glucose and galactose, which can then be absorbed across the intestinal epithelium (Figure 1). The lactase activity also cleaves other substrates (cellobiose, cellotriose, cellotetrose, and, to a certain extent, cellulose), while the phlorizin hydrolase activity splits beta-glycosides with large hydrophobic alkyl chains [7].

**Figure 2:**
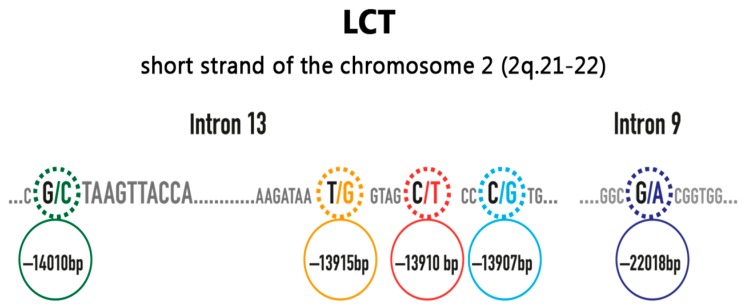
Some of the most important single nucleotide polymorphisms in the lactase gene (modified from [13]).

In Caucasian populations, the persistence or non-persistence of lactase expression is strictly associated with the single nucleotide polymorphism (SNP) C/T^-13910^ located upstream of the LCT-encoding gene (rs4988235) (Figure 2) [10]. This polymorphism emerges in the CC, CT, or TT variants [11]. It was demonstrated that in Caucasians, the CC variant is excellent as a predictor of the decline of intestinal lactase, and it is thus associated with hypolactasia, whereas the genotype TT is a predictor of the persistence of lactase activity. The presence of the CT genotype is characterized by the presence of intermediate levels of lactase expression, which are usually adequate for lactose digestion. As lactose malabsorption is a recessive condition, a heterozygous genotype has to be considered a negative test result [10]. It has been demonstrated that the majority of the lactase mRNA present in heterozygous individuals with persistent lactase activity originates from only one allele, which is consistent with their heterozygous status. This result is very informative, because it clearly demonstrates that adult-type hypolactasia is caused by a cis-acting transcriptional silencing of the lactase gene, and that the individual lactase alleles are regulated independently [12].

There are other polymorphisms that have been identified in the LCT gene and in surrounding regions, such as G/A^-22018^, which is linked to more than 95% of the non-persistence of lactase activity in the Finnish population, but routine tests are not widely available for these variants yet [9].

On the other hand, lactase persistence is mediated by several polymorphisms in different populations (G^-13915^ in Saudi Arabia and G^-14010^, G^-13915^, and G^-13907^ in African tribes); thus, lactase persistence seems to have developed independently in different areas of the world during human evolution (Figure 2) [6].

The lactase enzyme is shut off in a precisely timed developmental step. In this way, lactose malabsorption is useful in the promotion of weaning in the young and ovulation in the mother, and the lactose–lactase system can thus regulate optimal birth spacing in land mammals [4].

## 4. Hypolactasia, Lactose Maldigestion, Malabsorption, and Intolerance

The term hypolactasia refers to the deficiency of the lactase enzyme; this leads to lactose malabsorption, which is defined as an inefficient digestion of the disaccharide, which, in turn, can lead to lactose intolerance, a clinical condition defined as the presence of gastrointestinal symptoms due to lactose malabsorption (Table 1) [3,14].

Lactase deficiency exists as:

- Congenital lactase deficiency (alactasia), which is extremely rare, is due to the inheritance of 2 defective alleles of the LCT gene. The infant can suffer from watery diarrhoea after being fed with breast milk or food containing milk, and it can become a severe condition, as the shortage of nutritive ingredients can lead to growth delay, dehydration, and alkalosis; infants with congenital lactase deficiency were not expected to survive before the 20th century, when adequate lactose-free milk substitutes were not readily accessible [15]
- Primary lactase deficiency (adult-type hypolactasia) is caused by the non-persistence of lactase, with enzyme levels progressively reducing starting from the age of 2–5 years, depending on ethnicity [15]
- Secondary hypolactasia involves the loss of the lactase enzyme due to other clinical conditions affecting the intestinal tract. Since this enzyme is found on the apex of the duodenal villus, all pathological conditions involving the microvilli can result in the reduction of lactase. Once the primary problem is resolved, lactose-containing products can often be consumed normally. Clinical conditions leading to secondary hypolactasia include [5,15,16]:
  - severe malnutrition
  - celiac disease
  - inflammatory bowel diseases (Crohn’s disease, ulcerative colitis)
  - bacterial or viral enteritis (e.g., rotavirus), and parasitic disease (e.g., giardiasis, cryptosporidiosis)
  - actinic enteritis
  - some pharmacological treatments (kanamycin, neomycin, polymycin, tetracycline, colchicine, and other chemotherapeutic drugs)
  - some post-surgical conditions, such as stagnant loop syndrome or short bowel syndrome

## 5. Epidemiology of Lactose Intolerance

Genetically determined lactase enzyme non-persistence has a variable prevalence that strongly depends on the ethnic group, since it is much more prevalent in the Southern European population than in the Northern European population.

In Northern Central Europe, the lactase non-persistence phenotype is found in between 2% and 20% of the general population, while it accounts for approximately 40% of the population in Mediterranean countries (it is most common in Italy, where it is found, on average, in 56% of the population and where it is estimated to reach peaks of up to 70% in some regions), 65–75% in a large part of Africa, and up to more than 90% in Asia [17,18]. However, only individuals with non-persistent lactase show lactose intolerance symptoms and clinical signs [14].

However, surprisingly, considering the high prevalence of lactose deficiency in adulthood, even now lactose intolerance represents an under-diagnosed problem that is often examined after a marked delay compared to the onset of symptoms. This fact is even more surprising if compared to the attention paid to other conditions involving adverse reactions to food, such as coeliac disease and food allergies, that cumulatively affect merely 5% of the adult population overall.

## 6. Clinical Manifestations

As already stated, a deficiency of the lactase enzyme leads to lactose malabsorption, as the disaccharide cannot be absorbed in an undigested form and is fermented by gut microbiota [5]. This, in turn, leads to the development of symptoms that constitute the clinical condition of lactose intolerance.

In individuals with lactose intolerance, symptoms can be either gastrointestinal or extra-intestinal (Table 2) [3].

Gastrointestinal complaints that are almost invariably present in lactose-intolerant patients are diarrhoea, nausea, bloating, borborygmi, and abdominal pain [6]. The pathogenic mechanisms underlying these symptoms include abdomen distention caused by lactose fermentation by means of microorganisms in the intestinal gut flora and an osmotic effect produced by lactose molecules in the gastrointestinal tract [14]. It has also been hypothesized that a reduction of carbon dioxide to methane by some microbial strains could lead to constipation, which is reported by a minority of lactose-intolerant patients [14].

In many cases, extra-intestinal manifestations are also reported by lactose-intolerant patients, the most frequent of which are headache, asthenia, joint and/or muscle pain, loss of concentration, skin lesions, and mouth ulcers (Table 2) [3]. However, the existence of a lactose systemic syndrome is still controversial, and its pathogenic mechanism has not been clearly elucidated.

Since the symptoms, both gastrointestinal and systemic, can appear several hours after the consumption of food containing lactose, and since there is a wide distribution of lactose-containing products (even non-dairy products, as lactose is used as a food additive in different products), the lactose-intolerant patient is not always able to correlate the onset of these symptoms with the ingestion of lactose [19].

Moreover, the threshold of lactose tolerance varies significantly among patients and is dependent on several factors including the dose of lactose consumed, residual lactase expression, food matrix (ingestion with other dietary components), gut-transit time, and enteric microbiome composition [6]. Although lactase expression is not upregulated by lactose ingestion, it has been reported that regular intake of even small amounts of lactose may improve tolerance via adaptation of the intestinal flora [20].

## 7. Diagnosis

Originally, the diagnosis of hypolactasia in adulthood was based on the measurement of serum glycaemia 30 min after the consumption of 50 g of lactose; if the lactose was digested, and therefore absorbed, an increase of the glycaemic index to more than 20 mg/100 mL could be observed.

Later, measurement of the enzymatic activity in bioptic fragments of the small intestine mucous membrane was developed. Nevertheless, currently this technique is rarely used due to its invasive impact and high cost, as well as the fact that it can be influenced by the irregular distribution of lactase in the small intestine mucous membrane [12].

The hydrogen breath test (HBT) after the oral administration of lactose is currently considered the gold standard for lactose intolerance diagnosis due to its high sensitivity and specificity, its simplicity and non-invasiveness, and its low cost. It is based on the measurement of the quantity of hydrogen exhaled and recollected in samples every 30 min after the oral administration of lactose (usually 25 g, corresponding to approximately 500 mL of milk). The non-absorbed lactose in the colon is fermented by gut microbiota with the consequent production of hydrogen, which is partially excreted through the respiratory system. The HBT is positive when the hydrogen level in the exhaled air is at least 20 parts per million greater than the baseline value.

In previous studies, the HBT has shown a sensitivity of 76–100% and a specificity of 90–100% [21,22].

However, false negative results due to the lack of hydrogen production by colic bacterial flora or the recent administration of antibiotics and also false positive results due to the presence of bacterial overgrowth of the small intestine (SIBO) must be considered. To increase the accuracy of the test, it is recommended to avoid antibiotics in the four preceding weeks, to consume complex carbohydrates the preceding day, and to refrain from smoking and physical activity on the day of the test. Paying attention to a rapid increase of hydrogen (in the first 90 min from the beginning of the HBT) can help identify patients with SIBO [23].

The presence of subjective symptoms during the HBT can be useful for the confirmation of diagnosis, but it cannot replace the HBT, because when considered alone, the presence of symptoms has low sensitivity and specificity for the diagnosis of lactose intolerance [6]; at the same time, some patients do not associate their symptoms of lactose intolerance with the intake of lactose or—in many cases—with any kind of food [24].

For a differential diagnosis between primary and secondary hypolactasia, determining the presence of the SNP C/T^-13910^ is required; in the Caucasian population, the presence of this polymorphism can also be employed in lactose intolerance for diagnostic purposes, as it has shown marked sensitivity (97%) and specificity (95%) compared to those of the HBT [25].

For this reason, this kind of test carried out on the cleavage cells of the oral mucosa obtained by a simple swab (or even with a blood test) can be considered an alternative to the time-consuming and more challenging breath test in patients with gastrointestinal symptoms after the consumption of lactose-containing foods [25].

A comparison of diagnostic tests for lactose intolerance/malabsorption is shown in Table 3.

## 8. Management

The treatment for lactose intolerance mainly consists of reducing/eliminating the dietetic amount of lactose until the symptoms disappear. Most intolerant patients can tolerate 5 grams of lactose per single dose, with an increase in the tolerance threshold if the lactose is consumed together with other nutrients [20].

According to the European regulations for food labelling, the presence of milk or its derivatives—including lactose—should be reported on the label or in the ingredients list for freshly prepared products [26].

The absence in many European countries and non-European countries of laws regulating the commercialization of delactosed products—and the consequent lack of a cut-off value for establishing when a product can be labelled “lactose-free”—has resulted in the proliferation of many dairy products claiming the absence or reduction of lactose, despite the presence of a small amount (usually <0.01% or <0.1% and <0.5%, respectively) in such products, which, although reduced, is still enough to induce symptoms in at least a portion of lactose-intolerant patients.

In Italy, the lactose-intolerant patients’ association, AILI (Associazione Italiana Latto-Intolleranti), has recently asked the Italian Ministry of Health for the definitions of qualitative and quantitative standards for product labelling to be shared by producers, consumers, and institutions for the regulation of the “lactose-free” claim. Moreover, AILI has presented its report to the Environment, Public Health, and Food Safety Committee at the European Parliament in Brussels to encourage the definition of such standards for the whole European Union.

It must be emphasized, moreover, that lactose is also a widely used food additive (the so-called “hidden lactose”), which makes it even harder for patients to cope with this kind of intolerance, as lactose is frequently added to meats, frozen vegetables (including French fries), ready-made meals, sweets, and cakes.

In addition to dietetic restriction of lactose-containing foods, other approaches have been evaluated to reduce symptoms in these patients [20].

Lactase can be administered as an enzymatic food supplement, but its efficacy, of which convincing evidence is still lacking, is short-lived, and therefore, the supplement has to be consumed approximately 5–30 min before the lactose-containing meal [27,28].

It has been suggested that regular administration of increasing quantities of lactose may improve symptoms in lactose-intolerant patients via the mechanism of colonic adaptation [29,30]; however, very few studies have explored this approach, the evidence for which is scarce [20].

The non-absorbable antibiotic rifaximin has been evaluated in a single trial. After a course of 10 days, the lactose-related symptoms were reduced with respect to the baseline evaluation, reaching a level comparable to that after a 40-day lactose-free diet [31]; this result, however, has not been replicated in placebo-controlled trials or other independent studies.

### Probiotics as a Future Option in the Management of Lactose Intolerance

Recently, the use of specific probiotic strains, in particular those capable of expressing β-galactosidase enzymatic activity, has been proposed as an adjuvant treatment for subjects with lactose intolerance.

Probiotics are defined by the World Health Organization as live microorganisms, which, when administered in adequate amounts, confer a health benefit on the host [32]. Already, in 1974, it was suggested that fermented dairy foods could be beneficial for patients with lactose intolerance [33]. Some years later, it was discovered that lactase-containing microorganisms in yogurt and fermented milk could hydrolyse lactose [34], and today, the evidence that probiotics alleviate the clinical symptoms of lactose intolerance is increasing due to a growing number of relevant studies [35,36]. It is now clear, however, that the benefits that can be obtained depend a great deal on the species specificity (and in some cases the strain specificity) of the probiotic used [37,38].

In patients with irritable bowel syndrome (IBS), *Lactobacillus plantarum* has been tested in a four-week treatment and was shown to provide effective symptom relief, with particular efficacy against bloating and abdominal pain [39]. In the same context, *Lactobacillus acidophilus* was associated with reduced scores for abdominal pain and discomfort after a four-week treatment [40]. In an observational study with 96 adult IBS patients, a significant decrease in faecal calprotectin levels compared to the baseline was observed after two months of treatment with a multi-strain symbiotic (composed of *Bifidobacterium lactis* W51, *Lactobacillus acidophilus* W22, *Lactobacillus plantarum* W21, *Lactococcus lactis* W19, and inulin) [41].

In the case of lactose intolerance, the clinical benefit for the host derives from the β-galactosidase activity that strains of both *Lactobacillus* and *Bifidobacterium* have shown in preclinical and clinical settings [35,42,43,44]. A window of opportunity therefore exists to develop probiotic-containing foods and food supplements that can help ameliorate the symptoms of lactose intolerance, and this is considered one of the fields with the most supporting evidence and the strongest potential for the effective use of probiotics [45].

The results of the measurement of the β-galactosidase enzymatic activity of particular bacterial strains like those evaluated in a study [46] shown in Table 4 indicate that the production of β-galactosidase is highly strain-specific.

Modulation of the intestinal microbial environment by promoting intestinal colonization by strains capable of β-galactosidase activity could be an effective approach for the treatment of subjects with lactose intolerance. This could greatly improve the tolerance of small amounts of lactose (such as those often unknowingly consumed in non-dairy foods or those containing limited quantities of dairy products), with a prolonged effect (no need to take medications just prior to every meal) and with significant benefits for the patient’s quality of life.

Recently, a systematic review about the efficacy of several different probiotic strains in the management of lactose intolerance was published [48]; despite a limited number of reviewed studies (15), the improvement of intestinal conditions moving towards the amelioration of lactose intolerance has been demonstrated in several cases. The authors of that study, however, emphasized that variations in probiotic concentration, preparation, and β-galactosidase activity may affect the clinical efficacy of probiotic treatments [48].

## 9. Conclusions

Even if lactose intolerance has been recognized for 50 years, it still continues to be under-considered and under-diagnosed. Even if diagnosis can be challenging in some cases, both the HBT and the genetic tests (the latter in the Caucasian population) can be considered reliable and cost-effective tools to diagnose this condition.

Dietary restriction of lactose-containing foods is the main therapeutic intervention for lactose-intolerant people, but administration of lactase as enzymatic food supplement and of specific strains of probiotics expressing β-galactosidase activity can be of help in improving lactose tolerance and quality of life.

Since the current evidence for the effects of probiotics on lactose intolerance are still inconclusive, more studies with a careful selection of β-galactosidase-expressing strains are needed in order to explore their potential in this field.

## Figures and Tables

**Table 1:** Definition of hypolactasia, lactase non-persistence, lactose malabsorption, and lactose intolerance (modified from [14]).

**Hypolactasia**	Any deficiency of the lactase enzyme
**Lactase non-persistence**	This is considered the “wild-type” condition, as most individuals have reduced lactase activity at the jejunal border after weaning. In a minority of humans, a high level of activity of the lactase enzyme is present through all adulthood (lactase persistence phenotype)
**Lactose maldigestion**	Inefficient digestion of lactose, due to lactase deficiency (either lactase non-persistence or other intestinal conditions)
**Lactose malabsorption**	Inefficient absorption of lactose, due to lactose maldigestion, as lactose cannot be absorbed in the undigested form
**Lactose intolerance**	Gastrointestinal symptoms due to lactose malabsorption

**Table 2:** Most frequently reported gut-related and systemic symptoms in patients with lactose intolerance (modified from [3]).

Symptoms of Lactose Intolerance		Frequency (% of Total)
Gut-related symptoms	Abdominal pain	~100
	Gut distension	~100
	Borborygmi	~100
	Flatulence	~100
	Diarrhoea	70
	Constipation	30
	Nausea	78
	Vomiting	78
Systemic symptoms	Headache	86
	Loss of concentration	82
	Tiredness	63
	Muscle pain	71
	Joint pain/stiffness	71
	Mouth ulcers	30
	Increased frequency of micturition	<20

**Table 3:** Comparison of the characteristics of the tests currently available for assessing lactose malabsorption/intolerance (modified from [6]).

Summary of Available Tests for Assessing Lactose Malabsorption/Intolerance				
	Lactose Tolerance Test	H_2_-Breath Test (HBT)	Genetic Test	Lactose Activity at Jejunal Brush Border
Test principle	Increase of glycaemia after lactose challenge	Increase of H_2_ in expirate after lactose challenge	Assessment of 13910C/T polymorphism	Lactase enzymatic activity in bioptic sample
Cut-off criterion	<1.1 mmol/L within 3 h	>20 ppm within 3 h	C:C13910 Lactase non-persistence phenotype	<17–20 IU/g
Availability	Excellent	Good	Good	Rare
False positives	Rapid GI-transit, impaired glucose tolerance	Rapid GI-transit, SIBO	Rare (<5%) in Caucasians	Most likely, rare
False negatives	Fluctuations in glycaemia	Non-H_2_-producers, full colonic adaptation	All causes of secondary lactose malabsorption	Patchy enzyme expression
Secondary causes	Cannot be excluded	Cannot be excluded, kinetics of H_2_-increase can be suggestive	Cannot be excluded	Can be excluded (histopathology during same procedure)
Symptoms assessment	Possible	Possible	Not possible	Not possible
Cost	Lowest	Low	Medium	Highest
Comment	Low sensitivity and specificity	Method of choice for assessment of primary and secondary lactose intolerance	Method of choice for assessment of primary lactase deficiency in Caucasians	Invasive and expensive testing

**Table 4:** The β-galactosidase activity of several probiotic strains (modified from [46]).

Probiotic Strains	β-Galactosidase Activity Level
*Bifidobacterium lactis* W52	++++
*Bifidobacterium lactis* W51	+++
*Lactobacillus acidophilus* W22	+++++
*Lactobacillus acidophilus* W70	+++++
*Lactobacillus brevis* W78	+
*Lactobacillus casei* W20	+
*Lactobacillus casei* W79	++
*Lactobacillus plantarum* W21	+
*Lactobacillus rhamnosus* W71	+
*Lactobacillus salivarius* W24	+++++
*Lactococcus lactis* W19	+
*Streptococcus thermophilus* W69	+++++

Measurement of β-galactosidase enzymatic activity was performed, as described by Miller [47], by spectrophotometric measurement (405 nm) of the formation of the yellow chromophore ο-nitrophenol (ONP) as the hydrolytic product of the action of β-galactosidase on the colourless substrate o-nitrophenyl-β-galactoside. Higher levels of ONP indicate greater amounts of β-galactosidase released from the bacterial cells (+ to +++++).
